# Deletion of Glucocorticoid Receptors in Forebrain GABAergic Neurons Alters Acute Stress Responding and Passive Avoidance Behavior in Female Mice

**DOI:** 10.3389/fnbeh.2018.00325

**Published:** 2018-12-21

**Authors:** Jessie R. Scheimann, Parinaz Mahbod, Rachel Morano, Lindsey Frantz, Ben Packard, Kenneth Campbell, James P. Herman

**Affiliations:** ^1^Department of Pharmacology and Systems Physiology, University of Cincinnati, Cincinnati, OH, United States; ^2^Division of Developmental Biology, Cincinnati Children’s Hospital Medical Center, Cincinnati, OH, United States

**Keywords:** glucocorticoid receptor, GABAergic, sex differences, acute stress, passive avoidance

## Abstract

The glucocorticoid receptor (GR) is critically involved in regulation of stress responses [inhibition of the hypothalamic-pituitary-adrenal (HPA) axis], emotional behavior and cognition *via* interactions with forebrain corticolimbic circuity. Work to date has largely focused on GR actions in forebrain excitatory neurons; however, recent studies suggest a potential role mediated by interneurons. Here, we targeted GR deletion in forebrain GABAergic neurons, including the cortical interneurons, using a Dlx5/6-Cre driver line to test the role of forebrain interneuronal GR in HPA axis regulation and behavior. Our data indicate that GR deletion in GABAergic neurons causes elevated corticosterone stress responsiveness and decreased cross-over latencies in a passive avoidance task in females, but not males. Dlx5/6-Cre driven gene deletion caused loss of GR in interneurons in the prefrontal cortex (PFC) and hippocampus, but also in select diencephalic GABAergic neurons (including the reticular thalamic nucleus and dorsomedial hypothalamus). Our data suggest that GR signaling in interneurons is differentially important in females, which may have implications for GR-directed therapies for stress-related affective disease states.

## Introduction

The glucocorticoid receptor (GR) is critical for control of hypothalamic-pituitary-adrenal (HPA) axis reactivity, affective state and emotional memory in mice. Many mouse genetic knockdown/knockout (KD/KO) models have been created to elucidate the role of forebrain GR in these processes. In particular, numerous GR KO/KD models have been designed to assess the role of GR in potentiation of psychiatric illness and stress regulation. Pan-neuronal GR KOs (targeting all neurons and glia cells; GRNesCre) and forebrain glutamatergic neurons (FBGRKO) all increase anxiety-like and/or depressive-like behavior (Boyle et al., 2005, 2006; Furay et al., 2008; Hartmann et al., 2017; reviewed in Tronche et al., 1999; Wei et al., 2004; Kolber et al., 2008; Solomon et al., 2012), and most cause increased HPA axis responsiveness to stress (however see Vincent et al., 2013). Mice with overexpression of GR in the forebrain exhibit no changes in basal or stress induced corticosterone levels, but do show increased anxiety like behavior and increased immobility in the forced swim test (Wei et al., 2004).

Past studies of GR KO/KDs in mice have utilized promotors that primarily target excitatory neurons, using CaMKII-Cre mice as driver lines. The CaMKII-Cre line used generally directs Cre expression in glutamatergic neurons (although CaMKII-Cre lines can direct GR deletion to non-glutamatergic cells in regions such as the striatum and bed nucleus of the stria terminalis; Klug et al., 2012; Jennings et al., 2013; Wang et al., 2013). These data have led to the hypothesis that glucocorticoids are essential for regulating behavior and HPA axis function *via* modulating the activity of glutamatergic neurons in forebrain. However, little is known about how GR acts within interneurons. Evidence suggests that GR in interneurons of the prefrontal cortex (PFC) is lost after chronic stress concomitant with deficits in behavioral control (McKlveen et al., 2016). Indeed, Hartmann et al. (2017) demonstrate that selective deletion of GR in forebrain interneurons is not sufficient to modify behavior (in fear conditioning, elevated plus, and dark/light box) or HPA axis function in male mice. The role of GR in forebrain interneurons in the female brain has yet to be elucidated.

It is notable that the vast majority of GR KO/KD work has been performed in males. In the one study using both sexes, Solomon et al. (2012) demonstrated pronounced sex differences in the forced swim test (increased immobility) and anhedonia (decreased sucrose preference) in CaMKII-GR KO mice, with only males showing a behavioral phenotype. Moreover, unlike males, female CaMKII-GR KO mice do not show increases in basal or stress-induced secretion (Furay et al., 2008; Solomon et al., 2012). These data suggest that females are less affected by GR KO in excitatory glutamatergic neurons, and may thus employ alternative mechanisms of GR regulation.

In this study, we tested the hypothesis that GR regulates behavioral and HPA axis function *via* forebrain GABAergic neurons, including the cortical interneurons. To selectively KO the GR in forebrain interneurons, we generated GABAergic interneuron specific GRKD mice by breeding Nr3c1(GR)flox/flox mice with transgenic mice expressing Cre recombinase behind a Dlx5/6 promoter (Dlx5/6-Cre-IRES-EGFP; Stenman et al., 2003). These mice express Cre recombinase, and EGFP, in GABAergic interneurons that arise from the embryonic ganglionic eminences and migrate either in the rostral migratory stream (RMS) to the olfactory bulb or tangentially into the developing cerebral cortex (Stenman et al., 2003; reviewed in Marín and Rubenstein, 2001). In these studies, we demonstrate that deletion of the GR in forebrain interneurons causes HPA axis hyper-reactivity and deficits in passive avoidance behavior in females but not males, suggesting sex-specific engagement of interneurons vs. projection neurons in coordination of stress responses and emotional behavior.

## Materials and Methods

### Generation of Mice

CD-1 mixed background mice containing the Dlx5/6-Cre-IRES-EGFP transgene (Jackson Laboratories, Tg(mI56i-cre, EGFP)1Kc/J; Stenman et al., 2003), were bred with C57BL6/129 floxed mutant mice possessing loxP sites flanking exon 2 of the Nr3c1 gene (GR) GrloxP, Nr3c1flox; Brewer et al., 2003). This results in deletion of the floxed sequence of the Nr3c1 gene in the vast majority of forebrain interneuron populations of the offspring. We bred Dlx5/6-Cre+; Nr3c1 Het male mice offspring of the F1 generation with Nr3c1 Het female offspring of the F1 generation to produce littermate experimental mice with four different genotypes: Dlx5/6-Cre+; GR f/f; Dlx5/6-Cre-, GR f/f; Dlx5/6-Cre+ wt; and WT amongst F2 offspring. Dlx5/6-Cre+; GR f/f (Dlx5/6-GRKD) and Dlx-Cre-; GR f/f (Control) littermates were used for the behavioral and physiological measures. However, Dlx5/6-Cre+; wt littermates and Dlx5/6-Cre-; wt littermates were ran through the same assays alongside experimental animals to assess any effects of Cre expression, with no significant differences found between Dlx 5/6-Cre+ wt; Dlx5/6-Cre-; wt, Dlx5/6-Cre-; GR f/f mice (Supplementary Figure S1).

Two cohorts of mice were bred and ran through behavioral assays (Figure 4). Mice were group housed until the experiment began at 8 or 15 weeks and then single housed in pressurized intraventilated caging on a 12 h light/dark cycle with *ad libitum* access to food and water in a humidity and temperature controlled vivarium with males on one side of PIV rack, females on the other. Due to the individual filtered ventilation it is unexpected that scent and auditory cues could be transferred. Experiments occurred 1–6 h after housing room lights came on. We split mice into two cohorts so all mice would be at least 8 weeks of age at the start of behavioral testing, as to mitigate confounds of adolescents. As cohorts were subsequently, the age of the mice differed between cohorts. Mice in cohort 1 started the experiment at 9–10 weeks while mice in cohort 2 started the experiment at 15–16 weeks. Cohort 1 consisted of *n* = 7 female Dlx5/6 GRKD, *n* = 9 female Control, *n* = 11 male Dlx5/6 GRKD; *n* = 15 Control. Cohort 2 consisted of *n* = 10 female Dlx5/6 GRKD, *n* = 10 female Control, *n* = 9 male Dlx5/6 GRKD, *n* = 10 male Control. Female cycle was not monitored. All experimental procedures were conducted in accordance with the National Academy of Science’s Guide for the Care and Use of Laboratory Animals and were approved by the University of Cincinnati Institutional Animal Care and Use Committee.

**Figure 1:**
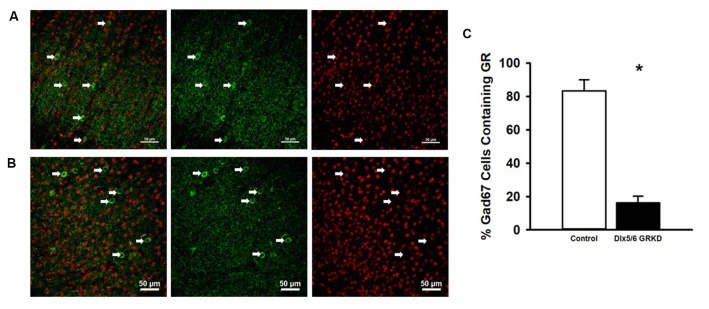
Dlx5/6 GRKD mice show glucocorticoid receptor (GR) knockdown (KD) in most Gad67+ cells. **(A)** Representative image of a control (Dlx5/6 Cre-; GR f/f) mouse prefrontal cortex (Prelimbic PFC) showing GR staining in Gad67 cells. **(B)** Representative image of a Dlx5/6 GRKD (Dlx5/6 Cre+; GR f/f) mouse PFC (Prelimbic PFC) showing little to no GR staining in Gad67 cells. **(C)** Dlx5/6 GRKD mice have significantly fewer cells with definitive GR staining in the Prelimbic. *p ≤ 0.05.

**Figure 2:**
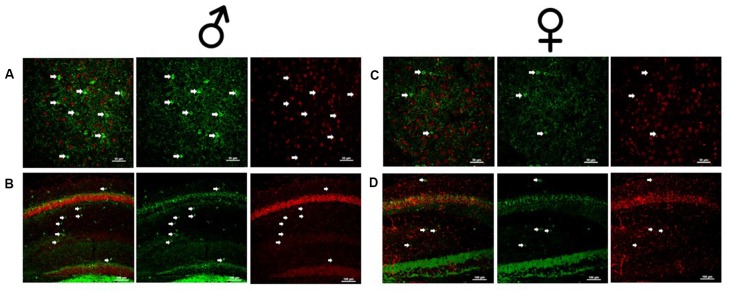
Male and female Dlx5/6 GRKD mice show KD of GR in corticolimbic regions. **(A)** Male basolateral amygdala. **(B)** Male hippocampus. **(C)** Female basolateral amygdala. **(D)** Female hippocampus.

**Figure 3:**
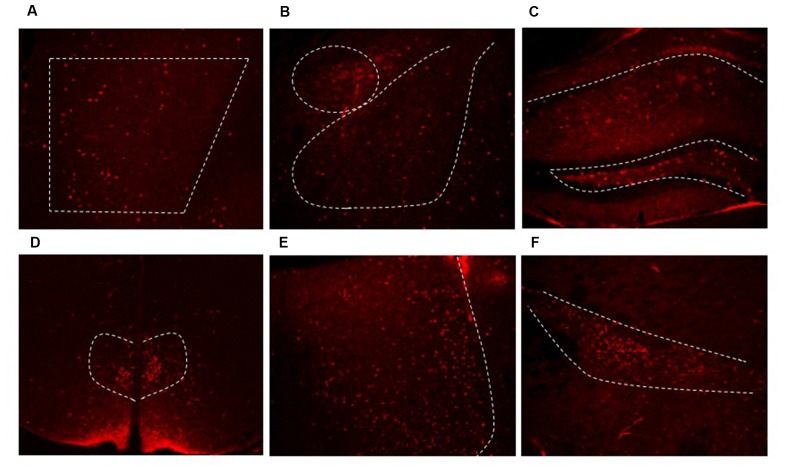
Dlx5/6 Cre-derived neurons are in areas throughout the forebrain. Representative images of Cre expression in the **(A)** PFC **(B)** amygdala **(C)** hippocampus **(D)** dorsal medial hypothalamus (DMH) **(E)** lateral septum **(F)** zona incerta.

**Figure 4:**
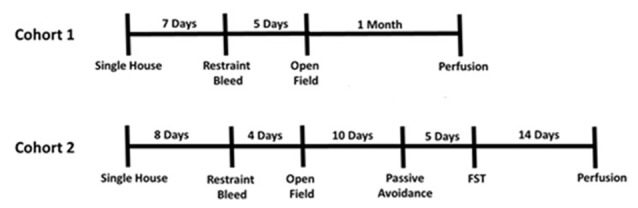
Experimental timeline.

### Restraint Stress Corticosterone Responses

Mice were restrained in well-ventilated 50 mL conical tubes for 30 min and then released into their home cage. Blood samples were taken from tail clip at the moment of restraint, 0 min, for basal levels, 15 min during restraint, 30 min during restraint (released from restrainers), 60 min, (freely moving in home cage), and 120 min (freely moving in home cage) from initiation of restraint. For tail clip at the 0 min time point, a sterile razor blade was used to nick less than 1 mm from the distal most part of the tail. Pressure was used to stop bleeding after each blood collection and a gentle rub with sterile gauze was used to remove the clot and reinitiate bleeding at subsequent time points. Plasma was kept on ice and immediately stored in −20°C after the experiment. Plasma samples were analyzed with I-125 radioimmunoassay (RIA) kit from MP Biomedicals. Data was pooled for each sex from both cohorts of mice.

### Behavior

For open field, mice were placed in a white plastic open field arena with an open top measuring 50 cm W × 50 cm L × 22 cm H under ambient room lighting. Mice were placed in a corner of the open field (consistent for all mice) and allowed to freely explore the arena for 10 min while being video recorded. Time the mouse spent in the center area (18.34% of the inner area) and total distance moved throughout the total arena was later analyzed with CLEVER software (CleverSys, Inc., Reston, VA, USA).

For forced swim testing, mice were placed gently into 2L beakers filled 3/4 full with tap water at 25°C (±2°C) for 10 min under ambient lighting. Mice were video recorded and later analyzed by hand by a blinded observer. The animal’s behavior, either swimming or immobile (defined as the minimum motion required for the mouse to keep afloat) were noted every 5 s and then calculated as % of total time bins.

For passive avoidance testing, on day 1, mice were placed in the lighted compartment of a light/dark shuttle box (San Diego Instruments, San Diego, CA, USA) with the center door closed. After 20 s the center door was raised manually and mice were free to enter the dark compartment. Once a mouse crossed all four paws over to the dark compartment, the door was manually closed and time to cross recorded. After a 2 s delay a 0.5 mA shock was administered for 2 s. Mice were removed from the dark compartment 30 s after the shock was administered back to their home cage. On day 2 (approximately 24 h after shock), mice were returned to the light compartment with the center door raised and time to cross to the dark compartment was measured. After crossing with all four paws to the dark compartment, the center door was closed and mice were removed 1 s later with no shock administered.

### Euthanasia and Organ Collection

Mice were injected with sodium barbital and transcardially perfused with 0.1 M PBS followed by 4% paraformaldehyde for 3–5 min. The thymus, heart, and adrenals were extracted and weighed after perfusion. Although differential changes in weight can be seen with perfusion, mice were given equal volumes of both PBS and paraformaldehyde. Brains were extracted and incubated in 4% paraformaldehyde for 24 h and then placed in 30% sucrose in PBS.

### Immunohistochemistry

Brains were sliced on a sliding microtome on a frozen stage (Leica Biosystems Inc., Nußloch, Germany) at 35 μm in series of 6. Slices were preserved in cryoprotectant (30% Sucrose, 1% Polyvinyl-pyrolidone (PVP-40), and 30% Ethylene glycol, in 0.1 M PBS) until assayed. For GR and glutamic acid decarboxylase 67 (Gad67) co-expression, slices were washed five times for 5 min (5 × 5) in PBS, and incubated in 1% sodium borohydride in PBS for 30 min, room temperature. Slices were washed 5 × 5 in PBS and then incubated in 1% H_2_O_2_ for 10 min at room temp. Slices were washed 5 × 5 in PBS and then incubated at 4°C overnight in blocking solution (4% goat serum, 0.2% BSA, 0.4% TritonX) with GR antibody (M20 Santa Cruz, SC-1004 rabbit) at 1:250 dilution and Gad67 (EMD Milipore, MAB5406 mouse) at 1:1,000 dilution. The following day slices were washed 5 × 5 in PBS and then incubated in secondary antibodies Cy3 anti-mouse (Invitrogen, Carlsbad, CA, USA, A32727) and cy5 anti-rabbit (Invitrogen, Carlsbad, CA, USA, A32733), both at 1:500 in blocking solution for 1 h at room temperature. Slices were washed 5 × 5 in PBS and mounted onto slices in PBS +1% gelatin.

For Cre Recombinase immunohistochemistry slices were washed 5 × 5 in PBS and blocked for 1 h room temp in 4% goat serum, 0.2% BSA, 0.4% TritonX. Primary incubations of Cre (EMD Millipore MAB 3120) occurred overnight at 4°C. On the following day, slices were washed 5 × 5 in PBS and incubated in Cy3 anti mouse (Invitrogen, Carlsbad, CA, USA, A32727) secondary for 1 h room temperature. Slices were then mounted in PBS +1% gelatin. Images were captured with a Nikon Confocal microscope (Nikon Instruments Inc. Melville NY, USA) at 10× to 20× objective. Basal Lateral Amygdala images were taken at an additional 1.55× zoom and the hippocampus at 1.6 zoom for clarity.

### Data Analysis

Sigma Plot/Sigma Stat 13.0 software (Systat Software, Inc.) was used to analyze all data. Data for weight and corticosterone measurements were analyzed with a two-way repeated measures analysis of variance (ANOVA). Fisher’s LSD was used for *post hoc* analysis for differences at individual time points. Data from both cohorts were pooled in respective groups for the corticosterone assay after a three-way repeated measures ANOVA (cohort × time × genotype) showed no significant effects between cohorts (*F*_(1,55)_ = 0.665; *p* = 0.418). Similarly, open field data was pooled as no significant cohort differences were evident (within cohort *t*-tests all showed *p* > 0.05). Due to the fact that the male data for the passive avoidance test did not pass equal variance and/or normality, both male and female data were analyzed with a Mann Whitney U non-parametric test (Rank Sum Test). Outliers were excluded from the analysis if they lied greater than 2 standard deviations from the mean. When data passed equal variance and normal distribution, parametric statistics were used. Male and female data were analyzed separately because female rodents naturally secrete higher corticosterone.

## Results

### Cre Recombinase Behind the Dlx5/6 Promotor Deletes GR in Interneurons

Due to the small, diffuse cell population of Dlx5/6-Cre neurons, we first assessed the level of GR KD in Dlx5/6 GRKD mice, using dual immunofluorescence. The Dlx genes (including Dlx5 and 6) are critical for migration of interneurons from the lateral ganglionic eminence to cortical regions (reviewed in Marín and Rubenstein, 2001), and given the previously reported lineage of Dlx5/6-derived neurons (Stenman et al., 2003) was thus expected to knock down GR in forebrain GABAergic neurons including the cortical interneurons. Figure 1 is a representative image taken in the prelimbic PFC showing GR staining present in Gad67+ neurons in a control (Dlx5/6-Cre-; GR f/f) mouse (Figure 1A) and GR KD in Gad67+ (a marker of GABAergic interneurons) cells (Figure 1B). We quantified the number of Gad67+ cells in the PFC of Dlx5/6 GRKD mice (Dlx5/6-Cre+; GR f/f, *n* = 6) and control (Dlx5/6-Cre-; GR f/f, *n* = 2) mice with GR KD. Due to the limitations of immunohistochemistry, complete KO cannot be assumed, and therefore cells without clear GR staining in the cell nucleus were considered KD (thus Dlx5/6 GRKD nomenclature will be used). We found the number of Gad67 cells containing GR in the cortex are significantly decreased in Dlx5/6 GRKD mice compared to control mice (*T*_(6)_ = 7.196; *p* < 0.001; Figure 1C). We took representative images of forebrain limbic structures important for HPA axis regulation and affective behaviors such as the BLA (Figures 2A,D) and the hippocampus (Figures 2B,C) to show that GR is knocked down in vast majority of Gad67 labeled neurons in both males and females.

From prior literature reports, we expected to see extensive Cre expression in interneurons in the forebrain. We used Cre expression remaining in adulthood as a marker for other regions where we may not have expected to see GRKD. Cre was found in limbic regions such as the PFC (Figure 3A), amygdala (Figure 3B), hippocampus (Figure 3C), the bed nucleus of the stria terminalis (not shown) and throughout the cortex. In addition, Cre expression was also found in numerous other GABA rich regions, including the dorsal medial hypothalamus (DMH; Figure 3D), lateral septum (Figure 3E), and zona incerta (Figure 3F). Cre expression was not observed in other areas rich in GABAergic neurons (e.g., peri-paraventricular nucleus, PVN region in the hypothalamus; Supplementary Figure S2). In addition, there was no evidence for colocalization of GR in Cre-positive neurons (data not shown).

### Male Dlx5/6 GRKD Mice Have Decreased Body Weight

**Figure 5:**
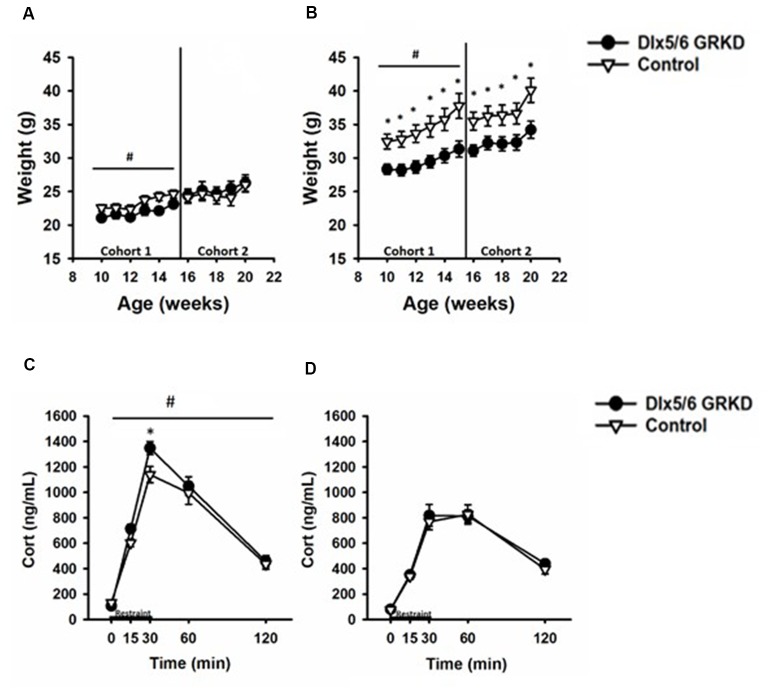
Dlx5/6 GRKD mice gain less weight than controls and female Dlx5/6 GRKD mice have higher peak Corticosterone (Cort) release to acute restraint. **(A)** Female Dlx5/6 GRKD mice showed a significant interaction between genotype and week in cohort 1, but no individual week was significantly different in *post hoc* analysis. Cohort 2 females only had a significant effect of time. **(B)** Male Dlx5/6 GRKD mice weighed less than the controls throughout the study. ^#^*p* ≤ 0.05 interaction; *p ≤ 0.05 *post hoc*. **(C)** Female mice showed an interaction between genotype and time during the acute restraint challenge. *Post hoc* analysis revealed cort was significantly higher for Female Dlx5/6 GRKD mice at the 30 min point (release point from restraint). **(D)** Male mice showed no differences in Cort throughout and after the restraint. ^#^*p* ≤ 0.05 interaction; *p ≤ 0.05 *post hoc*.

We measured weight weekly and found that female Dlx5/6 GRKD mice showed a significant interaction between genotype and week in cohort 1, but no individual week was significantly different in *post hoc* analysis (*F*_(4,60)_ = 2.645, *p* = 0.045). Cohort 2 females only had a significant effect of time (Time *F*_(4,60)_ = 13.635; *p* < 0.001; Figure 5A). Control male mice in cohort 1 weighed significantly more than Dlx5/6 GRKD mice [Cohort 1 (genotype × time *F*_(4,100)_ = 2.852, *p* = 0.02, Time *F*_(4,100)_ = 53.779, *p* < 0.001, genotype *F*_(4,100)_ = 6.625, *p* = 0.02)]. Cohort 2 mice only had a significant effect of time, no interaction (Time *F*_(4,100)_ = 43.042; *p* < 0.001; Figure 5B). Planned *post hoc* comparisons revealed a significant weight difference between male Dlx5/6 GRKD and controls at each week. Dlx5/6 may be expressed in other tissues of the body that are commonly affected by stress, such as the adrenals and thymus. Importantly, there were no differences in males nor females in weights of hearts, thymi, nor adrenals (Supplementary Figure S3).

### Female Dlx5/6 GRKD Mice Have Higher Peak Corticosterone Secretion Following Stress

Limbic forebrain regions including the PFC, amygdala and hippocampus are critical for negative feedback inhibition of the HPA axis after a psychological stress. These studies tested whether KD of GR in inhibitory neurons of the forebrain would cause similar, or perhaps opposing, effects on corticosterone. In females, there was a significant genotype by time interaction (*F*_(4,60)_ = 2.868, *p* = 0.032) in response to acute restraint challenge (Figure 5C). *Post hoc* analysis revealed corticosterone was significantly higher for Female Dlx5/6 GRKD mice at the 30 min point (release point from restraint; *p* < 0.05). (B) In contrast, male mice showed no differences in corticosterone during or after restraint (*F*_(4,100)_ = 0.372; *p* = 0.828; Figure 5D). Further, a three-way repeated measures ANOVA to compare males and females revealed a significant sex difference (*F*_(1,59)_ = 42.752; *p* ≤ 0.001). Female controls had higher corticosterone compared to male controls at the 15 min (*p* = 0.003), 30 min (*p* < 0.001), and 60 min (*p* = 0.022) time points. In Dlx5/6 GRKD mice females had higher corticosterone at the 15 min (*p* = 0.031), 30 min (*p* < 0.001), and 60 min (*p* < 0.001) time points. There was no sex × genotype interaction (*F*_(1,59)_ = 0.290; *p* = 0.592).

### Female Dlx5/6 GRKD Mice Show Decreased Latency in the Passive Avoidance Test

**Figure 6:**
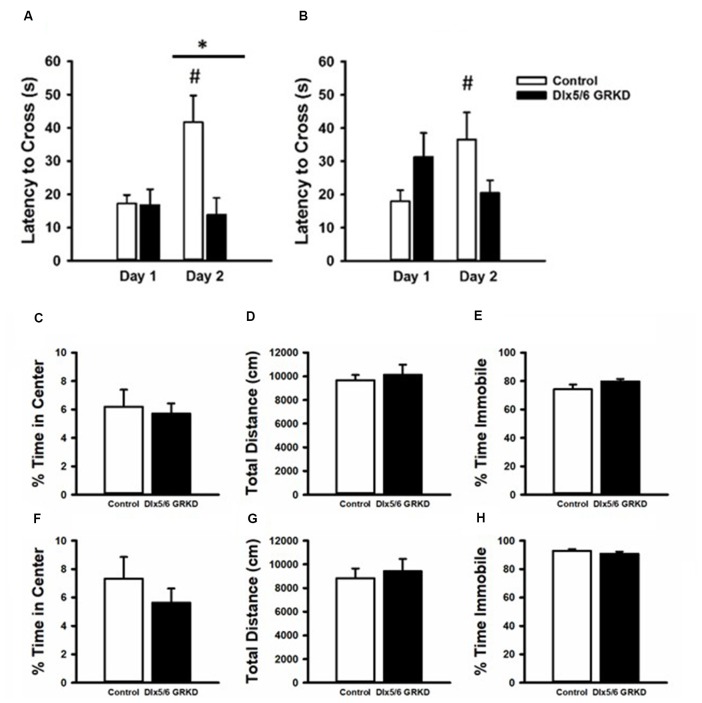
Passive Avoidance test of behavioral inhibition, forced swim test and open field. **(A)** Female mice showed no difference in latency to cross to the dark compartment on Day 1. While female controls learned and inhibited their behavior, female mice with Dlx 5/6 GRKD had decreased latency to enter the dark compartment compared to controls on day 2. **(B)** There was no difference in latency from controls in male Dlx 5/6 GRKD mice on day 1 or day 2. **(C–H)** Females Dlx 5/6 GRKD mice showed no differences in **(C)** center time or **(D)** total locomotion in the open field. Female Dlx 5/6 GRKD showed no differences in **(E)** immobility in the forced swim test. Similarly, male Dlx 5/6 GRKD mice showed no differences in **(F)** center time or **(G)** total locomotion in the open field, and immobility **(H)** in the forced swim test *p ≤ 0.05; ^#^*p* ≤ 0.05 for comparison of day within group.

To test if a change in behavioral control or impaired memory in an aversive context can be seen with GR loss in the forebrain Dlx5/6-derived neurons alone (in the absence of stress), we tested Dlx5/6 GRKD and control mice in the passive avoidance assay. Rodents have an inherent preference for dark confined spaces as opposed to open, bright areas that may carry environmental threats. Control mice will inhibit the compulsory behavior to escape to the dark compartment if previously given a foot shock in that chamber. A two-way repeated measures ANOVA revealed a day × genotype interaction for the females (*F*_(1,31)_ = 5.041; *p* = 0.041). On the first day, neither male nor female mice showed a difference between control and Dlx5/6 GRKD to enter the dark compartment before shock [Females: genotype within day 1 *p* = 0.968, Males: genotype within day 1 *p* = 0.204 (females Figure 6A; males Figure 6C)]. *Post hoc* analysis showed that female control animals took significantly longer to cross on day 2 (*p* = 0.006), whereas male Dlx5/6 GRKD have no significant change from day 1 to day 2 (*p* = 0.761). Female mice with Dlx5/6 GRKD had a lack of ability to inhibit cross over behavior, or were unable to recall the experience of shock in the dark compartment, as shown by a decreased latency to enter the dark compartment compared to controls on day 2 (*p* = 0.005; Figure 6A). There was no difference in latency on day 2 in male Dlx5/6 GRKD mice compared to controls (*p* = 0.062). Although male Dlx5/6 GRKD mice were not different from controls on day 2, male Dlx5/6 GRKD mice do not differ in time to cross from day 1 to day 2 (*p* = 0.408; Figure 6B). The lack of a significant difference between day 1 and day 2 suggests a deficit in learning or behavioral inhibition, although not definitive due to the lack of significant difference in genotype on day 2. While females seem to show deficits while males do not, no statistically significant sex differences were found using a three-way repeated measures ANOVA (Sex *F*_(1,33)_ = 1.024; *p* = 0.318, Sex × Day *F*_(1,33)_ = 0.046; *p* = 0.83, Sex × Genotype *F*_(1,33)_ = 0.506; *p* = 0.481) while the genotype × day effects still were present (*F*_(1,33)_ = 6.248; *p* = 0.017). However, the design of the experiment, with males ran on a different day than females, may not have been optimal for detection of statistical significant sex differences. Additionally, to see a statistical sex difference, females Dlx5/6 GRKD mice would have had to cross over to the dark compartment much faster than male mice with Dlx5/6 GRKD which was not observed.

We used the open-field assay to test for anxiety-like behavior. Mice will generally spend more time along the perimeter of the arena, and entry into the center is generally thought to represent decreased anxiety. Our male and female Dlx5/6 GRKD mice showed no differences from controls in center time (Males *T*_(17)_ = 0.296; *p* = 0.771; Females *T*_(17)_ = −0.995; *p* = 0.334; Figures 6C,F). There were no differences in distance traveled in the open field test in males or females (Males *T*_(17)_ = 0.139; *p* = 0.891; Females *T*_(17)_ = −0.0133; *p* = 0.990; Figures 6D,G). Finally, there were no differences observed in coping behavior in the forced swim test in either sex (Males *T*_(16)_ = 1.044; *p* = 0.312; Females *T*_(14)_ = −1.317; *p* = 0.104; Figures 6E,H).

## Discussion

Our study indicates that deletion of GR in populations of forebrain GABAergic neurons including the cortical interneurons resulted in HPA axis hyperactivity to an acute stress and impulsivity or impaired retention of passive avoidance learning in females. In contrast, male mice were minimally affected by interneuron GR deletion, showing no effects in behavior or stress responsiveness, only an attenuated body weight gain. The HPA axis and behavioral data suggest that in females but not males, HPA axis regulation and behavioral control in the passive avoidance test may be particularly vulnerable when GR is lost in inhibitory interneurons. The data suggest that GR signaling in interneurons may be disproportionately important in controlling stress responses and the ability to inhibit escape behavior in females.

Interneurons migrate tangentially to the forebrain during development from the subpallial telencephalon, *via* the medial and caudal ganglionic eminence. Many transcription factors are crucial for this migration including Dlx1, Dlx2, Dlx5, and Dlx6 (reviewed in Marín and Rubenstein, 2001). In this study, Dlx 5/6 was used as a promotor for Cre recombinase to KD GR in interneurons of the forebrain. By E12.5, the Dlx5/6-driven transgene is expressed throughout the medial and lateral ganglionic eminence including both the ventricular and subventricular zone of these mice, but decreases in expression toward birth (Stenman et al., 2003). Developmental decreases in Dlx5/6 transgene expression likely accounts for the relatively small proportion of cells that express Cre in adulthood, as opposed to the proportion showing GR KD (>80%). We also show that GR was deleted in Gad67 cells in both males and females in corticolimbic regions important for HPA axis regulation and affective behaviors. These data suggest that Cre protein expression during embryonic stages was sufficient to drive GR deletion in forebrain GABAergic neurons including cortical interneurons.

Despite not definitively marking interneurons with GRKD, Cre was expressed widely enough to be a marker for regions of the brain with Dlx5/6 targeted GR KD. As we know that interneurons from the ganglionic eminences can migrate to many forebrain regions, we used immunohistochemistry for Cre protein to track where we would expect GRKD. While the Dlx5/6-Cre-IRES-EGFP mice express GFP in addition to Cre, we were not able to detect GFP by constitutive activity or immunohistochemical staining, in agreement with a previous study that this transgene reporter is not highly expressed in adult brain (Allen et al., 2007). Using Cre staining as a surrogate, we observed expression in forebrain areas including the PFC, neocortex, hippocampus, central and basolateral amygdala. In addition, Dlx5/6 Cre+ neurons were found in additional brain regions, including the lateral septum, zona incerta, and the DMH. Note that regions showing Cre staining are rich in GABAergic neurons (RT, ZI), consistent with Dlx5/6 expression in inhibitory neuron populations beyond those destined for the cortex.

One region showing strong Dlx5/6-mediated Cre expression, and GR deletion, the DMH, may be involved in the body weight phenotype seen in male mice. The DMH regulates food intake. Excitotoxic lesions to the DMH produce hypophagia in rats (Bellinger et al., 1979) and attenuated weight gain following high fat diet (Bernardis and Bellinger, 1986). Reduced body weight is thought to be due to decreased activity of NPY neurons (a subclass of somatostatin interneurons) in the DMH (Bernardis and Bellinger, 1986). Hartmann et al. (2017) also report a lower body weight in the male Dlx5/6 GRKD mice. Reduced weight gain was not seen in Dlx5/6 GRKD females. It is possible that the lack of a body weight phenotype in females may be related to the general resistance of females to weight gain on a chow diet (Yang et al., 2014). Studies also suggest that ovariectomized females show increased adiposity in rats that is attenuated by DMH lesions (Bernardis et al., 1981), raising the possibility that female gonadal hormones may inhibit weight gain *via* interactions with GR.

Hartmann et al. (2017) recently published findings using Dlx5/6 GRKD mice. In agreement with our data, there was no effect of Dlx5/6 GRKD in male mice on HPA axis reactivity or behavior. However, females were not included in this study. Deletion directed toward forebrain projection (putative glutamatergic neurons; CaMKII GRKD) in males caused exaggerated HPA axis responses to restraint. Moreover, CaMKII GRKD causes increased anxiety-related behavior in the light-dark box and elevated plus maze, as well as deficits in auditory fear extinction, again, in males. Notably, prior work using CaMKII GRKD mice found pronounced sex differences in HPA reactivity and behavior, with physiological and behavioral effects observed only in males (Boyle et al., 2006; Furay et al., 2008; Solomon et al., 2012). Our data suggest that in females, glucocorticoids modulate stress responses and behavioral inhibition by altering inhibitory neuron function including the cortical interneurons, rather than excitatory circuitry. In combination, the data suggest that there are pronounced sex differences underlying forebrain mechanisms of stress regulation and emotionality, with interneurons playing a more prominent role in females.

The source of the sex difference remains to be determined. Females and males both show robust KD of GR in GABAergic neurons in key corticolimbic regions. Estrogen receptor alpha and beta is found in corticolimbic interneurons, thus making it possible that gonadal steroids may preferentially modulate sensitivity to GR in females (Blurton-Jones and Tuszynski, 2002). It is also possible that Dlx5/6 GRKD may alter estrogen signaling in interneurons, as glucocorticoids have been shown to inhibit estrogen responses and drive expression and activity of estrogen sulfotransferase (SULT1E1), an enzyme that deactivates estrogen receptor function, *via* reduced estrogen binding to the receptor (Gong et al., 2008). Sex differences may also be related to GR effects on gonadal steroid signaling, organizational impact of gonadal steroids on GR signaling in males vs. females, and perhaps even processes signaling sex differences in interneurons. These possibilities will need to be addressed in future studies.

In females, deletion of GR in interneurons decreased cross-over latency in the passive avoidance task but did not significantly impact other behavioral endpoints. In the passive avoidance task, the mouse learns that it must inhibit its innate preference to escape a slightly noxious environment after receiving a more aversive stimulus in the “safe” area the day before. The test therefore combines emotional learning and contextual fear conditioning which rely heavily on limbic regions for processing (Thompson, 1978; Ogren and Stiedl, 2010). The passive avoidance deficits in our females may be due to an inability to learn that the dark side contains a noxious stimulus (perhaps associated with GR deletion in the hippocampus and amygdala, or PFC). For instance, Roozendaal et al. (1993) found that lesion of CEA produce deficits in acquisition of the aversion in the passive avoidance. Additionally, Roozendaal et al. (2006) found that GR agonist infused into the PFC enhanced memory of the task, while antagonist decreased performance, indicating a role of GR (however, not cell type specific) in the task. In this regard, it is notable that the Dlx5/6 promoter will direct deletion to CeA neurons (Waclaw et al., 2010), which may account at least in part for altered avoidance behavior in females.

Another interpretation of the passive avoidance test is that the animal has an inability to inhibit the behavior of escaping to the dark even after receiving the noxious stimulus previously (perhaps associated with GR deletion in the PFC; Ogren and Stiedl, 2010; Camp et al., 2012). More testing, perhaps in non-aversive protocols like the 5-choice serial reaction time test or delayed spatial win-shift, would be required to demonstrate an “impulsivity” like phenotype.

These data reveal that inhibitory neuron (e.g., cortical interneuron) GR is required for normal regulation of stress reactivity and behavioral inhibition in females. Males appear to require GR in excitatory neurons for stress and emotional regulation as KO in CaMKIIα neurons produce higher corticosterone and deficits in fear conditioning, elevated plus, and dark/light box, forced swim test, and sucrose preference (Boyle et al., 2005, 2006; Furay et al., 2008; Solomon et al., 2012; Hartmann et al., 2017). These data point to a pronounced sex difference in glucocorticoid-mediated stress regulatory mechanisms, and suggest that mechanisms controlling stress processing, and by extension, stress-related diseases differ at a fundamental neurocircuit level in the two sexes. These data have important implications for treatment of stress-related disease, as it will become important to consider the possibility that male and female stress reactivity and emotional behavior are mediated by distinct neurocircuit mechanisms.

## Author Contributions

JS conceptualized and designed the study, acquired the data, analyzed the data, interpreted data and drafted the manuscript. PM and RM bred mice, acquired data, contributed to the design. LF acquired data and assisted in data analysis. BP acquired data and contributed to execution of assays. KC critically revised the manuscript for intellectual content. JH conceptualized study, interpreted data, and critically revised the manuscript for intellectual content for the final approval of the version to be published.

## Conflict of Interest Statement

The authors declare that the research was conducted in the absence of any commercial or financial relationships that could be construed as a potential conflict of interest.
