# Supplementary material for: Deletion of Glucocorticoid Receptors in Forebrain GABAergic Neurons Alters Acute Stress Responding and Passive Avoidance Behavior in Female Mice
Source: Front Behav Neurosci. 2018 Dec 21;12:325. doi: 10.3389/fnbeh.2018.00325 (PMC6309161; doi:10.3389/fnbeh.2018.00325)

Figure S1. Weights, corticosterone and passive avoidance for all genotypes showing no effect of Cre Recombinase. A) Females in cohort 1 showed no interaction with week an genotype [$F(18,138) = 1.610$; $p = 0.065$] or genotype alone [$F(3,138) = 2.405$; $p = 0.093$]. Cohort 1 females only showed an effect of week [$F(6,138)=18.799$; $p<0.001$]. Similarly, females in cohort 2 showed an effect of week [$F(4,64)$; $p<0.001$], but no effect of genotype [$F(2,64) = 0.984$; $p=0.395$] and no interaction effect [$F(8,64)=1.572$; $p = 0.151$]. Cohort 1 Dlx5/6 GRKD n=10; Control n=10; Dlx5/6Cre+ WT n=3; Dlx5/6Cre-WT n=4. Cohort 2 Dlx5/6 GRKD n=7; Control n=9; Dlx5/6Cre+ WT n=5; There were no Dlx5/6 Cre-WT in cohort 2. B) Males in cohort 1 showed an interaction between week and genotype [$F(15,105) = 1.833$; $p = 0.039$] and an effect of week [$F(5,105) = 39.699$; $p<0.001$]. B) Males in cohort 1 did not show an effect of genotype [$F(3,105) = 2.717$; $p=0.071$]. Cohort 2 males did not show a genotype by week interaction [$F(12,100) = 1.309$; $p=0.225$], but did show an effect of time [$F(4,100) = 18.634$; $p <0.001$] and genotype [$F(3,100) = 2.986$, $p = 0.50$]. # = interaction effect $p <.05$. Fisher's LCD test revealed a significant difference between only Dlx5/6 GRKD and Controls at each week. C) Corticosterone after acute restraint (cohorts pooled together). Females only showed an effect of time when all genotypes were analyzed: genotype [$F(3,219) = 1.847$; $p = 0.154$], time [$F(4,219)=132.071$; $p <0.001$], Genotype x time [$F(12,219) = 1.243$; $p =0.258$]. Dlx5/6 GRKD n=17, Control n= 18, Dlx5/6 Cre-WT n=4, Dlx5/6 Cre+WT n=3. D) Males also only showed an effect of time when all genotypes were analyzed genotype [$F(3,272) = 2.488$; $p=0.071$, Time $F(4,272) = 60.971$; $p<0.001$; genotype x time $F(12,272) = 0.381$; $p=0.969$]. * Fishers LCD – 30 min Dlx5/6 GRKD vs. Dlx5/6 Cre- WT $p=0.005$, Control vs Dlx5/6 Cre- Wt $p=0.036$. Dlx5/6 GRKD n=22, control n=24, Dlx5/6 Cre+WT n=5, Dlx5/6 Cre-WT n=5. E) Day 2 of Passive avoidance showing no effect of genotype. Females had a significant interaction effect [$H=7.389$; $p = 0.025$] with Dunn's posthoc showing only Dlx5/6 GRKD and Controls being different [$p= 0.020$] and no differences with Cre+ Wt animals. Dlx5/6 GRKD n= 6; Control n=10; Dlx5/6 Cre+WT n=5. E) F) Males showed on differences in day 2 of passive avoidance [$H=5.584$; $p = 0.061$].

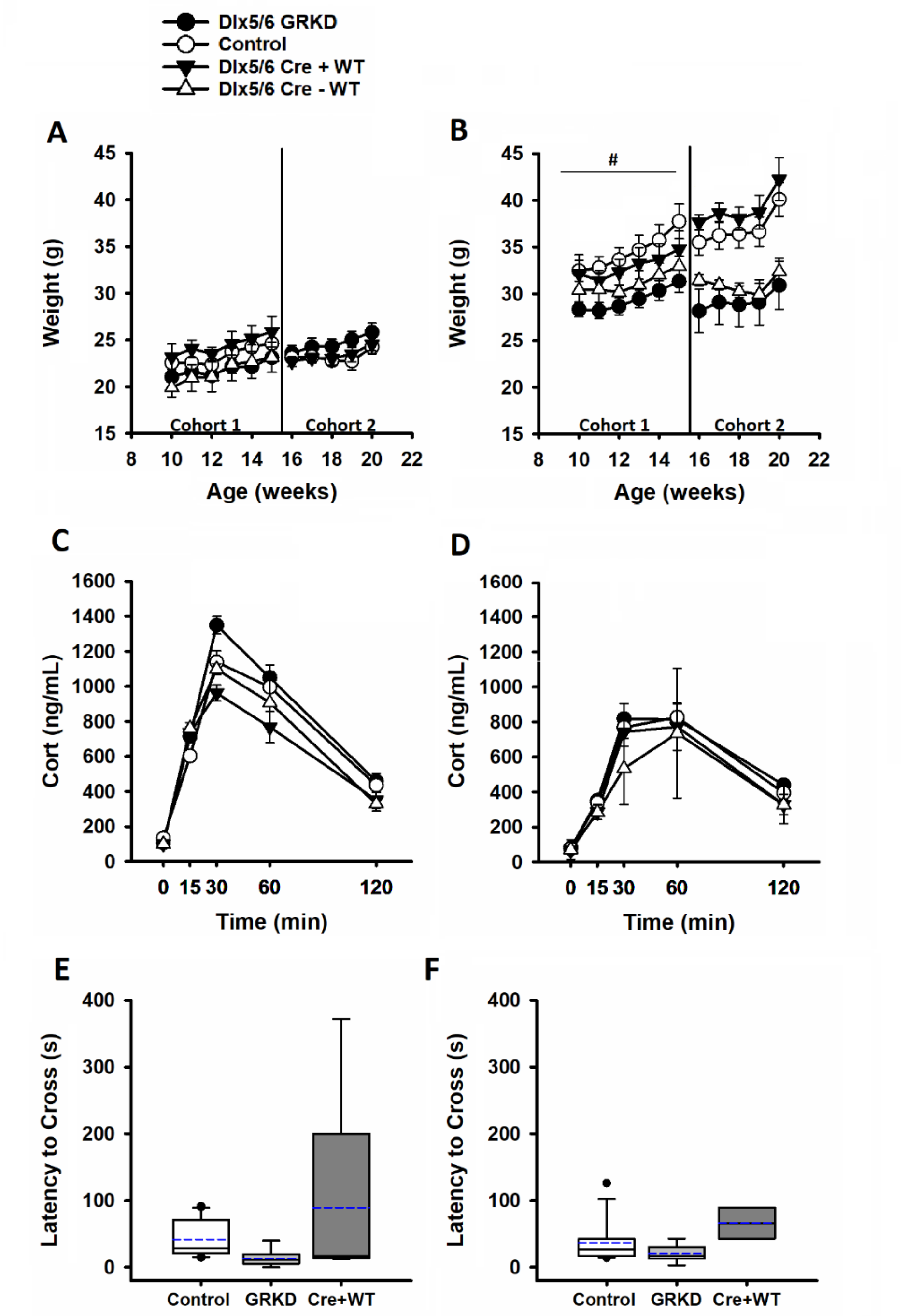

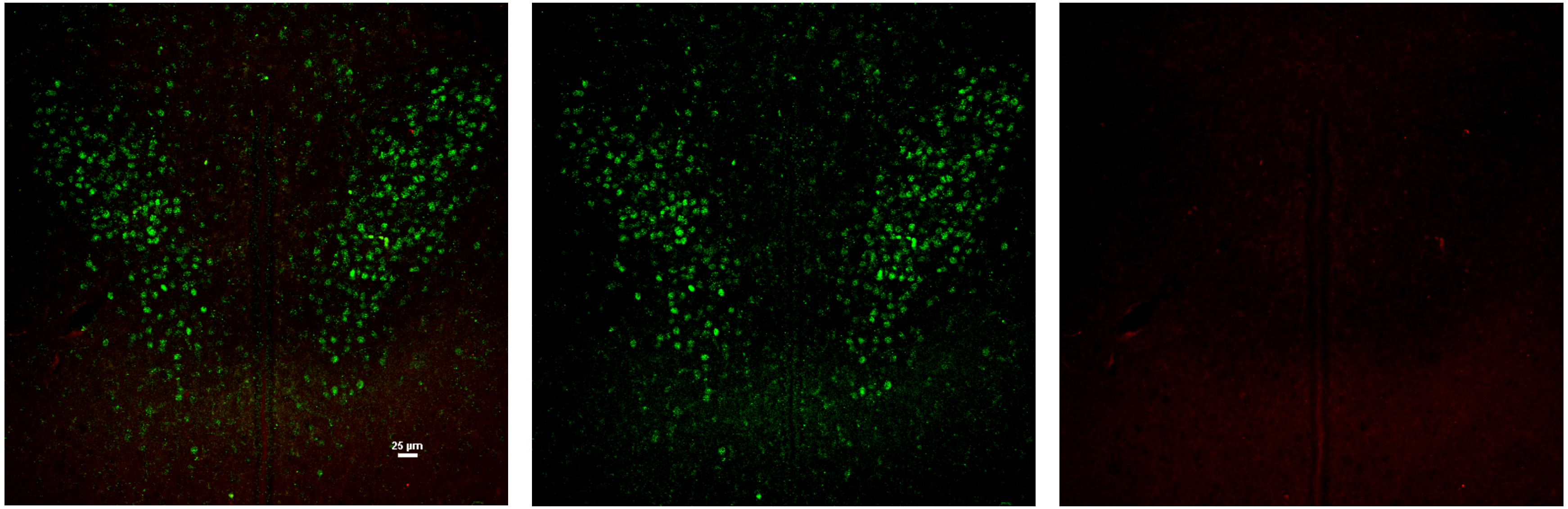

Figure S2. Representative image showing no Cre staining and robust GR expression in the paraventricular nucleus of the hypothalamus (PVN) (red=Cre, Green=GR).

Figure S3. Dlx5/6 GRKD organ weight does not differ from controls A) Female hearts [t(17) = 0.451; p = 0.658] B) Male hearts [t(17) = -1.727; p = 0.102] C) female Thymus [t(14) = .466 ; p = 0.466] D) male thymus [t(18) = 0.604; p = 0.553] E) female adrenals (averaged) [t(17)= -0.998 ; p = 0.332] F) male adrenals (averaged) t(17) = -0.685 ; p = 0.502].

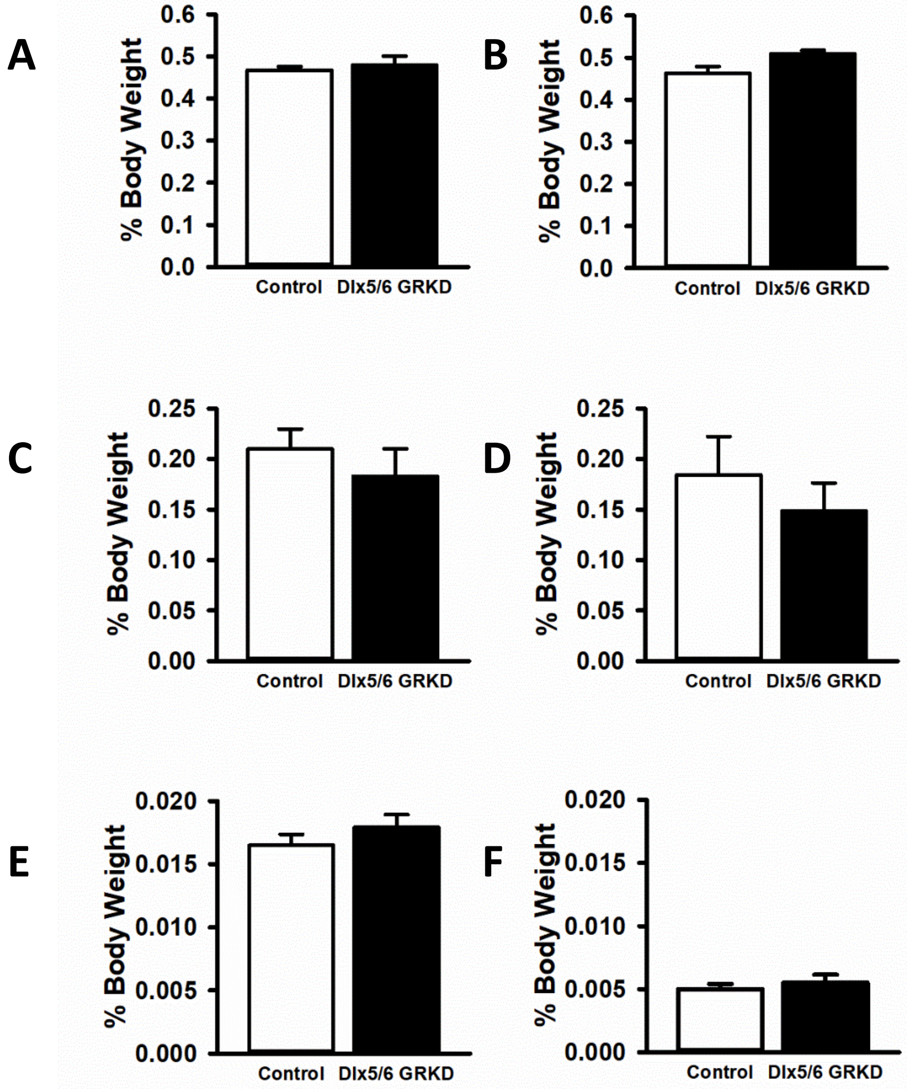

Supplement: Supplementary file 1 [file Presentation_1.pdf]
